# Tortuosité veineuse rétinienne idiopathique bilatérale de découverte fortuite

**DOI:** 10.11604/pamj.2024.48.180.44655

**Published:** 2024-08-16

**Authors:** Daouda Konaté, Bréhima Mariko

**Affiliations:** 1 Centre hospitalier Universitaire Bocar Sidy Sall (CHU-BSS), Institut d’Ophtalmologie, Kati, Mali,; 2 Centre Hospitalier Universitaire, Institut d´Ophtalmologie Tropicale de l´Afrique (CHU-IOTA), Bamako, Mali

**Keywords:** Tortuosité veineuse, bilatérale, idiopathique, Venous tortuosity, bilateral, idiopathic

## Abstract

Retinal venous tortuosity is characterized by an abnormally twisted presentation of the retinal veins. It may be associated with multiple retinal diseases. The main differential diagnoses include hypertensive retinopathy, which can lead to venous tortuosity, hemorrhages and exudates, diabetic retinopathy, where venous tortuosity occurs due to chronic retinal ischemia and chronic decreased retinal blood flow syndrome, which can cause generalized venous dilation. We here report an isolated case of bilateral retinal venous tortuosity of unknown origin and without visual consequences, discovered incidentally during a routine fundus examination in a 24-year-old young man with no known pathological history. A family clinical investigation involving five family members across three generations did not reveal similar cases, as this rare clinical presentation has often been described as hereditary. The diagnosis of idiopathic bilateral retinal venous tortuosity was retained. Cardiovascular examination was normal. Ophthalmological management involved regular monitoring of visual acuity and fundus. During the last check-up (three weeks ago), we observed that the veins remained tortuously dilated without functional impact, with visual acuity 10/10 P2 in both eyes.

## Image en médecine

La tortuosité veineuse rétinienne est caractérisée par une présentation anormalement sinueuse des veines de la rétine. Elle peut s´associer à diverses pathologies médicales de la rétine. Les principaux diagnostics différentiels sont: la rétinopathie hypertensive qui peut entrainer des tortuosités veineuses, des hémorragies et des exsudats; la rétinopathie diabétique ou la tortuosité veineuse est présente en raison de l´ischémie rétinienne chronique et le syndrome de bas débit rétinien chronique qui peut provoquer une dilatation veineuse généralisée. Nous rapportons un cas isolé de tortuosité veineuse bilatérale de la rétine de cause inconnue et sans conséquence visuelle découvert par hasard lors d´un examen systématique du fond d´œil chez un jeune homme de 24 ans sans antécédant pathologique connu. L´enquête clinique familiale réalisée sur cinq membres de la famille appartenant à trois générations différentes n´a pas retrouvé des cas similaires puisque cette présentation clinique rare a souvent été décrite comme héréditaire. Nous avons retenu le diagnostic de tortuosité veineuse rétinienne idiopathique bilatérale idiopathique. L´examen cardio vasculaire demandé s´est révélé normal. La prise en charge ophtalmologique a consisté en une surveillance régulière de l´acuité visuelle et du fond d´œil. Lors du dernier contrôle il y a trois semaines nous avons constaté que les veines restent tortueuses dilatées sans impact fonctionnel avec une acuité visuelle 10/10 P2 aux deux yeux.

**Figure 1:**
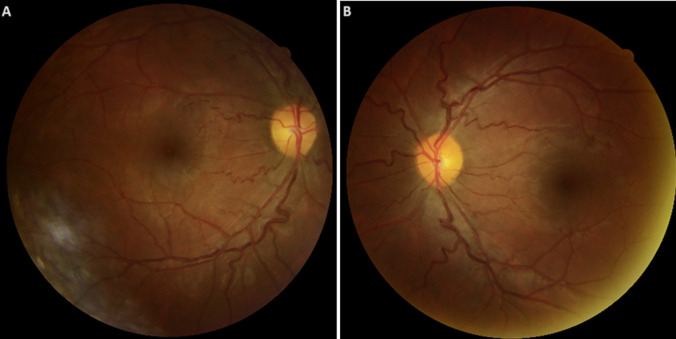
A, B) rétinographie bilatérale avec des veines rétiniennes dilatées et tortueuses

